# AI Health Literacy: a reflective framework for LLM-based generative AI in public health education and promotion

**DOI:** 10.3389/fpubh.2026.1891658

**Published:** 2026-07-20

**Authors:** Viviane Scherenberg

**Affiliations:** Department of Public Health and Environmental Health, APOLLON University of Applied Sciences, Bremen, Germany

**Keywords:** AI Health Literacy, digital health equity, eHealth literacy, generative AI, health literacy, large language models (LLMs), public health

## Abstract

Generative artificial intelligence (AI) is changing how citizens search for, understand, and use health information. Large language models (LLMs) can simplify, translate, summarize, and tailor health-related content to individual questions. At the same time, AI-generated responses may appear fluent, plausible, and empathic without necessarily being complete, up to date, evidence-based, or suitable for a person's individual situation. This creates a public health challenge for Public Health Education and Promotion, as AI-generated answers may increasingly shape how citizens access, interpret, and trust health information. Building on health literacy, eHealth literacy, digital health literacy, and debates on AI-mediated health communication, this article conceptualizes AI Health Literacy as a task- and context-sensitive extension of existing literacy concepts. Its specific contribution is to understand AI Health Literacy as an interdisciplinary judgement competence that requires citizens to assess not only the content of AI-generated health information, but also the task, context, modality, verifiability, and psychological conditions under which such information is received and used. It proposes a reflective framework with three analytical dimensions: task appropriateness, context of use, and critical verifiability. The central argument is that generative AI can support understanding, orientation, translation, and preparation, but should not replace professional advice in diagnostic, therapeutic, triage-related, medication-related, or crisis situations. The framework aims to support public-health-oriented education, communication, and institutional guidance for the responsible use of AI-generated health information and should be understood as a conceptual orientation tool, not as a validated assessment instrument.

## 1 Introduction

Generative AI is becoming increasingly relevant to public health because it can make health information more accessible, easier to understand, and more responsive to individual questions. Large language models (LLMs) can explain medical terms, simplify complex texts, summarize information, translate content, and help citizens prepare questions for medical consultations. These functions may be particularly useful for people who struggle with complex health information or who need guidance in uncertain situations (1, 2). At the same time, LLMs are changing the logic of health information seeking. Traditional search engines usually provide multiple results, websites, and sources, whereas LLMs generate conversational and coherent answers. These answers may appear complete and personally relevant, even though users often cannot immediately judge how accurate, current, evidence-based, or appropriate they are for their own situation (1, 3). This development should be situated within the broader shift from intermediation and apomediation to AI-mediated health communication. Eysenbach conceptualized apomediation as a model in which users access and assess health information through networked guidance rather than only through traditional intermediaries (4). In AI-mediated environments, by contrast, generative systems may select, synthesize, prioritize, and frame information linguistically in a single conversational response. Romero-Rodriguez and Castillo-Abdul describe this transition as AImediation and argue that generative AI can reconfigure informational authority in health communication (5). This argument is extended by research showing that AImediation also reconfigures patient autonomy as an outcome shaped by the interaction between algorithms, interfaces, and information-saturated cognitive environments (6). This concern relates to the ethical problem described by Grote and Berens, whereby algorithmic decision-support systems may create an “illusion of autonomy” by preserving the appearance of individual choice while shaping the options, framings, or recommendations on which those choices are based (7). This matters because studies and reviews of generative AI in healthcare point to a range of risks, including hallucinations, bias, privacy concerns, limited transparency, and answers that are difficult to verify (8–10). From a public health perspective, this raises the question of under what conditions citizens can interpret, critically evaluate, and responsibly use AI-generated health information. This is where AI Health Literacy becomes relevant. In this article, AI Health Literacy is defined as the ability to understand AI-generated health information, assess its evidential quality and limitations, judge the appropriateness of AI use for a specific health-related task and context, recognize uncertainty and risk, and seek professional or institutional support when the situation requires it. The specific contribution of this article is to conceptualize AI Health Literacy as an interdisciplinary judgment competence that extends beyond the evaluation of AI-generated content alone. Citizens must assess not only the content of AI-generated health information, but also the task, context, modality, verifiability, and psychological conditions under which such information is received and used. So far, the evidence on AI and health literacy remains limited and methodologically heterogeneous. A systematic review indicates that existing studies examine different AI applications, target groups, research questions, and health literacy outcomes, and often do not directly involve users (11). This article therefore does not claim to validate a new measurement scale or assessment instrument. Rather, it offers a conceptual framework that may serve as a starting point for future empirical research, public health education, communication, and institutional guidance. Against this background, this article asks which competencies citizens need when dealing with AI-generated health information. It focuses on when generative AI may support understanding and guidance, and when more careful assessment, attention to the context of use, and professional advice become important.

## 2 Conceptual foundations and distinctiveness of AI Health Literacy

Health literacy is widely understood as more than an individual skill, shaped by personal competencies, information environments, institutional communication, and social conditions (12, 13). The model by Sørensen et al. describes health literacy as the ability to access, understand, appraise, and apply health information (14). eHealth literacy extends this perspective to digital information environments and emphasizes the ability to search for, understand, evaluate, and use digital health information (15). AI Health Literacy builds on these concepts but adds a specific focus on AI-generated health information. Users still need the core abilities associated with health literacy, including the ability to understand, appraise, and apply health information. They also need the digital skills emphasized in eHealth and digital health literacy, because AI outputs are accessed through digital platforms, devices, and interfaces. LLMs change this starting point because users no longer simply retrieve information; they interact with systems that generate, reformulate, and adapt answers in real time. This creates additional requirements for AI Health Literacy. Citizens need to recognize that AI-generated answers may be incomplete, inaccurate, outdated, insufficiently traceable, or unsuitable for their individual situation. They also need to distinguish decision-related recommendations from general guidance and assess when professional advice is needed. These requirements presuppose general health literacy, since users must be able to appraise AI-generated health information, assess risks, recognize warning signs, and relate statements to their own situation. AI Health Literacy therefore does not replace health literacy or eHealth literacy; rather, it builds on both and extends them to AI-supported information environments. AI Health Literacy thus involves more than technical skills. It is not enough to know how to write a prompt or use a chatbot. What matters is the ability to interpret AI-generated health information in a task-specific, critical, and context-sensitive way. This is particularly relevant because, for example, ChatGPT-generated medical responses may appear useful, while their information quality, traceability, and alignment with clinical guidelines remain limited or uncertain (16). The framework was developed by integrating four thematic areas of literature: health literacy, eHealth literacy, and digital health literacy; generative AI in healthcare and psychological aspects of information perception and processing; apomediation, AImediation, misinformation, and informational authority; and digital health equity, trust, vulnerability, and contextual factors. Together, these areas informed the distinction of AI Health Literacy from related concepts, the three dimensions of the framework, and the discussion of practical implications. As this is a Perspective article, the framework should be understood as a conceptually derived analytical structure rather than a comprehensive review of all relevant research.

## 3 A reflective framework for AI Health Literacy

**Figure 1:**
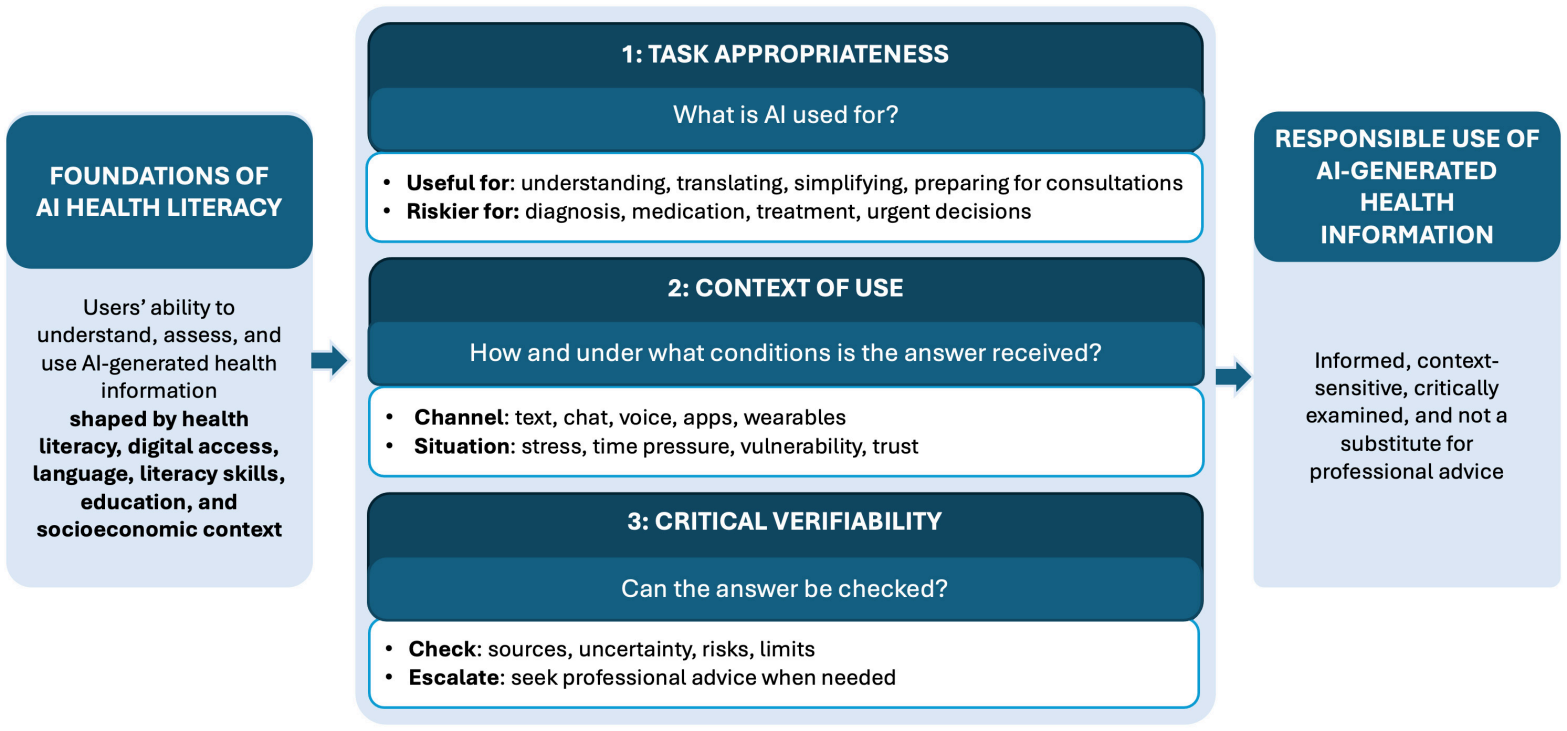
Reflective framework for AI Health Literacy in Public Health.

The reflective framework translates the requirements described in Section 2 into three analytical dimensions: task appropriateness, context of use, and critical verifiability (Figure 1). It asks what AI is being used for, through which channels and formats citizens receive AI-generated answers, and how these answers can be critically examined. The aim of the framework is to provide a public-health-oriented structure for assessing when AI-generated health information may support understanding and preparation, and when stronger verification, professional advice, or institutional safeguards become necessary. Trust, vulnerability, equity, and institutional responsibility are understood as cross-cutting conditions because they influence who uses AI, how answers are processed, and who can benefit from AI-supported health information. The three dimensions were selected because they correspond to three sequential questions that arise whenever citizens use generative AI for health-related information. First, what is the purpose of use? This is addressed by task appropriateness. Second, under which situational, social, and technical conditions is the output received and interpreted? This is addressed by context of use. Third, can the output be checked, challenged, and connected to trustworthy sources or professional advice? This is addressed by critical verifiability. Other aspects, such as trust, vulnerability, equity, and institutional responsibility, are not excluded; rather, they are treated as cross-cutting conditions that shape all three dimensions.

### 3.1 Dimension 1: task appropriateness

The first dimension addresses task appropriateness, that is, whether generative AI is suitable for the specific health-related purpose for which it is used. Generative AI may be useful for understanding, translating, or simplifying health information and preparing for consultations. Its use becomes riskier, however, when questions involve symptoms, diagnoses, medication, treatment decisions, urgent-care decisions, or acute health concerns. In such cases, the suitability of an AI-generated answer also depends on whether users are able to describe their situation sufficiently. Prompting skills as well as reading and writing skills play a role, as does the ability to describe one's own situation accurately and adequately. Incomplete or unclear inputs can lead to general answers that may nevertheless influence health-related actions. Reviews of LLMs in patient care indicate that rapidly available answers may be incomplete, inaccurate, or difficult to verify (8, 17). Findings from non-health-specific studies also suggest that AI may be more useful for supportive tasks than for decision-making tasks. The benefit of AI support also depends on whether users can interpret the answers in light of relevant knowledge and critically evaluate them (18). For AI Health Literacy, this means that citizens need to be able to assess how close their use of AI is to a health-related decision. They need to distinguish whether they are using AI to better understand information, prepare for a consultation, or inform a health-related decision. The closer an AI-generated answer comes to a health-related decision, the more important source checking, awareness of uncertainty, and professional advice become. This is particularly true for diagnostic questions, as findings on diagnostic performance show that generative AI should not replace professional assessment (19).

### 3.2 Dimension 2: context of use

The second dimension focuses on the context of use. It asks under which media-related, technical, social, emotional, linguistic, and institutional conditions AI-generated health information is received and interpreted. What matters is therefore not only the content of an answer, but also the channel, device, interface, and presentation format through which it reaches users. Generative AI no longer appears only in chat windows. It is increasingly embedded in search engine summaries, voice assistants, apps, and wearables. These formats influence how AI-generated answers can be processed and checked. Text-based answers can be reread more easily and compared with sources, whereas spoken answers are more transient and require users to rely more heavily on memory when evaluating them. In a study on text- and audio-based health information, Leroy and Kauchak found that text and audio produced partly comparable results for immediate comprehension, while text showed advantages for recall and reproduction (20). A meta-analysis on reading and listening comprehension further shows that differences between the two modalities depend on content, task, and complexity (21). Literacy is therefore an important condition for processing health information. Text-based AI-generated answers may be easier to retrieve and verify, but they require sufficient reading and writing skills. Auditory or dialogic AI-generated answers may be more accessible for people with lower literacy, but, based on evidence from health literacy, eHealth literacy, and digital inequality research, they may also be most transient and therefore harder to review later (19, 21, 22). In addition to channel, device, and interface, the specific situation of use is important. Citizens often seek health information not under neutral conditions, but when they are worried, under time pressure, experiencing acute symptoms, or facing upcoming health decisions. Such situations influence how AI-generated answers are understood and used. An answer may then be perceived not only as preliminary guidance, but also as a reason to wait, self-treat, or seek professional help. Stress, anxiety, prior knowledge, language, illness experience, social support, and trust in the healthcare system are discussed in research on digital health literacy, digital inequality, and health communication as relevant influencing factors (23, 24). AI Health Literacy therefore requires situational self-assessment: users should ask not only whether an answer sounds clear, but also whether their current stress, vulnerability, language barriers, digital access, or trust in the healthcare system may affect how they formulate prompts, interpret outputs, and use AI-generated information. For AI Health Literacy, this means that not only the AI-generated answer itself must be assessed, but also the context in which it is received, understood, and potentially used to guide action.

### 3.3 Dimension 3: critical verifiability

The third dimension focuses on critical verifiability. It asks whether an AI-generated output is not only understandable, but also sufficiently transparent and checkable. This distinction is important because AI Health Literacy goes beyond mere comprehension. Citizens must not only understand what an AI-generated answer says, but also be able to assess what it is based on, what uncertainties exist, and whether professional advice is needed. A central difficulty is that an answer can sound clear and accessible without being medically reliable. Clarity, empathy, and linguistic fluency can be helpful, but they do not replace information quality, medical safety, or a traceable evidence base (1, 19). For users, this can be particularly challenging because linguistically convincing answers may foster trust. Ethical analyses of LLMs in medicine also emphasize risks related to biased training data, limited transparency, and potentially misleading content (25). The QUEST framework, an evaluation framework for LLM responses in healthcare, also shows that comprehensibility alone is not a sufficient quality criterion. The quality of the information, the reasoning behind the answer, potential risks, and the trustworthiness of the response must also be assessed. QUEST describes these requirements through the dimensions Quality of Information, Understanding and Reasoning, Expression Style and Persona, Safety and Harm, and Trust and Confidence (26). Empirical studies support this caution. In comparisons with static online information, ChatGPT showed useful features but also limitations regarding information quality and alignment with clinical guidelines (16). In relation to vaccine myths, answers were often understandable and correct, but could still be misleading when used by laypeople (27). Positive ratings of empathy and response quality primarily show how an AI-generated answer is perceived. They do not automatically demonstrate medical appropriateness or suitability for health-related decisions (28). Critical verifiability is therefore not only a task for users. It also depends on whether AI-generated answers are designed in a way that makes assessment possible in the first place. This includes verifiable sources, recognizable uncertainties, clear boundaries, indications of missing evidence, and clear advice on when professional consultation is necessary. Without these elements, an answer may appear understandable without being sufficiently verifiable.

### 3.4 Trust, vulnerability, and equity as institutional conditions

For public health, the relevant question is not only what AI systems can do, but also how trust, vulnerability, and equity shape the use of AI-generated health information. Public perceptions of AI in healthcare are differentiated. Fritsch et al. found that many respondents viewed AI in medicine positively in principle, while still considering medical supervision important (29). Older people, women, individuals with lower educational levels, and people with lower affinity for technology were more cautious. Rojahn et al. also report that trust, cultural assumptions, and medical oversight influence public evaluations of AI in healthcare (30). Trust is therefore ambivalent. Too little trust may prevent people from using helpful support functions. Too much trust may lead users to adopt AI-generated answers uncritically. A systematic review on trust in digital healthcare shows that trust may be influenced by perceived risk, data privacy, data quality, digital competence, form of interaction, education, and income (31). Public health education should therefore aim for calibrated trust: citizens should be able to use AI when it is helpful, but also recognize when caution and professional advice are necessary. Equity is equally important. Digital health literacy is socially unequally distributed and includes several subcompetencies (22). Digital literacy is also discussed as a determinant of health because it may influence access, self-management, and participation in health decisions (23). Studies on eHealth interventions further show that socially disadvantaged groups are not always adequately considered (32). For AI-generated health information, this creates a double public health challenge. On the one hand, LLMs may lower barriers by simplifying, translating, explaining information conversationally, or making it available in audio form (1, 2). On the other hand, new inequalities may emerge if those who benefit most are primarily people who can formulate precise prompts, compare answers, check sources, and recognize uncertainty. People with low literacy, language barriers, limited digital participation, or low trust in digital systems may be at greater risk of using AI-generated answers uncritically or inappropriately. This assumption should not be understood as a confirmed AI-specific effect, but rather as a conceptual transfer from research on digital health literacy, mHealth, and digital inequality (23, 32, 33).

## 4 Implications for public health education and promotion

Public health education should not reduce AI Health Literacy to technical tool use. It is not enough to show citizens how to operate a chatbot. More important is the ability to distinguish appropriate from inappropriate uses. Three competencies are central. First, citizens need to assess the task: Am I using AI for understanding, preparation, or decision support? Second, they need to recognize uncertainty: Does the answer provide sources, limitations, and alternatives? Third, they need to recognize when advice is needed: When is a professional necessary? Education should not promote maximum skepticism, but calibrated trust. Citizens should be able to benefit from AI-supported explanation, translation, and preparation without trusting AI blindly. This is particularly important for vulnerable groups. Digital tools for informed decision-making among vulnerable screening invitees are helpful only when they are adapted to preferences, information needs, and support requirements (34). Reviews on AI and diverse or marginalized populations also indicate that insufficient representation in data and development contexts may reinforce inequalities (35). Institutions also bear responsibility. Health insurers, healthcare providers, public health services, and educational organizations should provide clear guidance on when AI use for health questions is appropriate and where its limits lie. The framework is therefore relevant not only for citizens, but also for health educators, clinicians, health communication professionals, public health institutions, insurers, digital health designers, and policymakers. In Germany, this is particularly relevant for statutory health insurance funds, which are required under § 20k SGB V to provide services that promote the self-determined and health-oriented use of digital or telemedical applications and procedures (36). Possible applications include citizen-facing checklists, training modules, school or university health literacy curricula, community education materials, consultation preparation tools, and institutional guidance on appropriate and inappropriate AI use. Research on digital health equity suggests that digital services need to be designed in an understandable way, supported during onboarding, embedded in trustworthy contexts, and implemented in a target-group-sensitive manner (32, 33). Table 1 translates the proposed reflective framework into practical assessment questions for public health education and health promotion. It should be understood as a conceptually derived guidance tool rather than a validated instrument.

**Table 1:** Practical assessment questions derived from the reflective framework for AI Health Literacy.

Domain	Assessment question for users	Warning sign	Recommended interpretation	Appropriate AI use	Inappropriate AI use
Task	*Am I using AI only to understand something, or to support a decision?*	The answer recommends waiting, self-treatment, or concrete action	In decision-related situations, involve professional advice	Understanding, translating, or simplifying health information; preparing questions for a consultation	Using AI to diagnose symptoms, choose a treatment, change medication, or assess acute complaints
Health-related risk	*Does the question involve symptoms, medication, diagnosis, therapy, or crisis?*	Acute symptoms, psychological crisis, medication, or emergency relevance	Do not use AI as the sole basis for decision-making	Using AI for general orientation or to prepare a conversation with a health professional	Using AI-generated advice as a basis for self-diagnosis, self-treatment, or delaying professional help
Sources and uncertainty	*Does the answer provide verifiable sources and limitations?*	No sources, overly confident language, no indication of uncertainty	Check, compare, and contextualize the answer	Checking sources, comparing the answer with trusted health information, and considering uncertainty	Accepting unverifiable or source-free answers because they sound clear, certain, or reassuring
Situation of use	*Am I under stress, anxiety, or time pressure?*	The answer is used alone, at night, or in acute worry	Do not act hastily; seek support	Using AI when there is time to reflect on the answer, compare it with other information, and ask for clarification if needed.	Relying on AI in acute, stressful, or time-critical situations without professional support
Comprehensibility	*Do I really understand the answer?*	Technical terms remain unclear or the answer only appears simple	Ask for clarification or simplification, but verify the content	Asking AI to explain terms, simplify information, or help formulate questions	Treating a fluent or empathic answer as medically reliable without critically questioning it
Advice	*Should I speak with a professional?*	The question concerns diagnosis, therapy, medication, triage, or crisis	Prioritize medical, psychosocial, or other professional advice	Using AI to prepare for a consultation or clarify questions for a health professional	Replacing medical, psychological, pharmaceutical, or emergency advice with AI-generated information
Access and barriers	*Could language-related, digital, or social barriers limit use or verification?*	Uncertainty when formulating the question, limited digital participation, language barriers	Provide low-threshold, multilingual, and non-digital support	Using AI together with support, trusted sources, multilingual materials, or guidance from health professionals or institutions	Using AI answers for health-related decisions when language, understanding, or opportunities for verification are limited

## 5 Discussion and conclusion

AI Health Literacy should be understood as a population-level, task- and context-sensitive extension of existing health literacy concepts. The focus is not the technology itself, but how citizens use AI-generated health information in everyday situations. The proposed reflective framework identifies three central questions. First, what is AI being used for? Second, through which channels and in which situations do users receive and process AI-generated answers? Third, is the answer merely understandable, or is it also verifiable? This perspective is relevant for public health because citizens often use health information tools when they are uncertain, worried, or under time pressure. In such situations, fluent AI-generated answers can provide guidance, but they may also create false reassurance. The current evidence on AI Health Literacy remains limited and often relies on artificial test questions, selected versions of AI models, specific medical topics, or English-language responses. Therefore, it remains insufficiently understood how different population groups understand, evaluate, and use AI-generated answers in real-life health information situations (1, 11, 37). Several limitations should be acknowledged. The proposed framework is conceptual and has not been empirically validated; it should therefore not be interpreted as a measurement instrument or as evidence that the proposed dimensions predict safe AI use. No original empirical data were collected, and the literature selection was purposive rather than systematic, so relevant studies may have been missed. In future research, the framework may need to be adapted to newer, multimodal, voice-based, or embedded AI systems, as well as to different languages, cultures, healthcare systems, levels of digital access, and degrees of institutional trust. AI Health Literacy is therefore not only an individual competence, but also a responsibility of public health institutions, education systems, and digital information environments. As healthcare systems and digital health technologies become more complex, foundational health literacy becomes even more important, because citizens need to understand information, assess risks, navigate services, and make informed decisions. Citizens need access to AI, but they also need guidance, safeguards, and equitable conditions for responsible use.

## Data Availability

The original contributions presented in the study are included in the article/supplementary material, further inquiries can be directed to the corresponding author.

## References

[1] Pal A Wangmo T Bharadia T Ahmed-Richards M Bhanderi MB Kachhadiya R . Generative AI/LLMs for plain language medical information for patients, caregivers and general public: opportunities, risks and ethics. Patient Prefer Adherence. (2025) 19:2227–49. doi: 10.2147/PPA.S527922 40771655 PMC12325106

[2] Tilton AK Caplan BE Cole BJ. Generative AI in consumer health: leveraging large language models for health literacy and clinical safety with a digital health framework. Front Digit Health. (2025) 7:1616488. doi: 10.3389/fdgth.2025.1616488 40933812 PMC12417475

[3] Wardle C Urbani S Wang E. Evolving health information-seeking behavior in the context of Google AI Overviews, ChatGPT, and Alexa: interview study using the think-aloud protocol. J Med Internet Res. (2025) 27:e79961. doi: 10.2196/79961 41055948 PMC12541266

[4] Eysenbach G. From intermediation to disintermediation and apomediation: new models for consumers to access and assess the credibility of health information in the age of Web2.0. Stud Health Technol Inform. (2007) 129:162–6. doi: 10.3233/978-1-58603-773-4-162 17911699

[5] Romero-Rodriguez LM Castillo-Abdul B. From Apomediation to AImediation: Generative AI and the Reconfiguration of Informational Authority in Health Communication. J Prim Care Community Health. (2025) 16:21501319251381878. doi: 10.1177/21501319251381878 40990570 PMC12461036

[6] Ponce-Rojo A Fontaines-Ruiz T Romero-Rodríguez LM. Apomediation, AI, and the illusion of autonomy: risks of misinformation in patient decision-making. Front Commun. (2026) 10:1684370. doi: 10.3389/fcomm.2025.1684370

[7] Grote T Berens P. On the ethics of algorithmic decision-making in healthcare. J Med Ethics. (2020) 46:205–11. doi: 10.1136/medethics-2019-105586 31748206 PMC7042960

[8] Moulaei K Yadegari A Baharestani M Farzanbakhsh S Sabet B Afrash MR. Generative artificial intelligence in healthcare: a scoping review on benefits, challenges and applications. Int J Med Inform. (2024) 188:105474. doi: 10.1016/j.ijmedinf.2024.105474 38733640

[9] Sallam M. ChatGPT utility in healthcare education, research, and practice: systematic review on the promising perspectives and valid concerns. Healthcare (Basel). (2023) 11:887. doi: 10.3390/healthcare11060887 36981544 PMC10048148

[10] Templin T Perez MW Sylvia S Leek J Sinnott-Armstrong N. Addressing 6 challenges in generative AI for digital health: a scoping review. PLOS Digit Health. (2024) 3:e0000503. doi: 10.1371/journal.pdig.0000503 38781686 PMC11115971

[11] Abeo ANA Armstrong S Scriney M Goss H. Artificial intelligence techniques and health literacy: a systematic review. Mayo Clin Proc Digit Health. (2025) 3:100269. doi: 10.1016/j.mcpdig.2025.100269 41211528 PMC12589913

[12] Nutbeam D Lloyd JE. Understanding and responding to health literacy as a social determinant of health. Annu Rev Public Health. (2021) 42:159–73. doi: 10.1146/annurev-publhealth-090419-102529 33035427

[13] Sørensen K Levin-Zamir D Duong TV Okan O Brasil VV Nutbeam D. Building health literacy system capacity: a framework for health literate systems. Health Promot Int. (2021) 36:i13–23. doi: 10.1093/heapro/daab153 34897445 PMC8672927

[14] Sørensen K Van den Broucke S Fullam J Doyle G Pelikan J Slonska Z . Health literacy and public health: a systematic review and integration of definitions and models. BMC Public Health. (2012) 12:80. doi: 10.1186/1471-2458-12-80 22276600 PMC3292515

[15] Norman CD Skinner HA. eHealth literacy: essential skills for consumer health in a networked world. J Med Internet Res. (2006) 8:e9. doi: 10.2196/jmir.8.2.e9 16867972 PMC1550701

[16] Walker HL Ghani S Kuemmerli C Nebiker CA Müller BP Raptis DA . Reliability of medical information provided by ChatGPT: assessment against clinical guidelines and patient information quality instrument. J Med Internet Res. (2023) 25:e47479. doi: 10.2196/47479 37389908 PMC10365578

[17] Busch F Hoffmann L Rueger C van Dijk EH Kader R Ortiz-Prado E . Current applications and challenges in large language models for patient care: a systematic review. Commun Med. (2025) 5:26. doi: 10.1038/s43856-024-00717-2 39838160 PMC11751060

[18] Vaccaro M Almaatouq A Malone T. When combinations of humans and AI are useful: a systematic review and meta-analysis. Nat Hum Behav. (2024) 8:2293–303. doi: 10.1038/s41562-024-02024-1 39468277 PMC11659167

[19] Takita H Kabata D Walston SL Tatekawa H Saito K Tsujimoto Y . A systematic review and meta-analysis of diagnostic performance comparison between generative AI and physicians. NPJ Digit Med. (2025) 8:175. doi: 10.1038/s41746-025-01543-z 40121370 PMC11929846

[20] Leroy G Kauchak D. A comparison of text versus audio for information comprehension with future uses for smart speakers. JAMIA Open. (2019) 2:254–60. doi: 10.1093/jamiaopen/ooz011 31294421 PMC6603442

[21] Clinton-Lisell V. Listening ears or reading eyes: a meta-analysis of reading and listening comprehension comparisons. Rev Educ Res. (2022) 92:543–82. doi: 10.3102/00346543211060871

[22] Diviani N van den Putte B Giani S van Weert JCM. Low health literacy and evaluation of online health information: a systematic review of the literature. J Med Internet Res. (2015) 17:e112. doi: 10.2196/jmir.4018 25953147 PMC4468598

[23] Dratva J Schaeffer D Zeeb H. Digitale Gesundheitskompetenz der Bevölkerung in Deutschland: aktueller Stand, Konzepte und Herausforderungen. Bundesgesundheitsblatt Gesundheitsforschung Gesundheitsschutz. (2024) 67:277–84. doi: 10.1007/s00103-024-03841-5 38315221 PMC10927882

[24] Arias López MDP Ong BA Borrat Frigola X Fernández AL Hicklent RS Obeles AJT . Digital literacy as a new determinant of health: a scoping review. PLOS Digit Health. (2023) 2:e0000279. doi: 10.1371/journal.pdig.0000279 37824584 PMC10569540

[25] Haltaufderheide J Ranisch R. The ethics of ChatGPT in medicine and healthcare: a systematic review on large langu age models. NPJ Digit Med. (2024) 7:183. doi: 10.1038/s41746-024-01157-x 38977771 PMC11231310

[26] Tam TYC Sivarajkumar S Kapoor S Stolyar AV Polanska K McCarthy KR . A framework for human evaluation of large language models in healthcare derived from literature review. NPJ Digit Med. (2024) 7:258. doi: 10.1038/s41746-024-01258-7 39333376 PMC11437138

[27] Deiana G Dettori M Arghittu A Azara A Gabutti G Castiglia P. Artificial intelligence and public health: evaluating ChatGPT responses to vaccination myths and misconceptions. Vaccines. (2023) 11:1217. doi: 10.3390/vaccines11071217 37515033 PMC10386180

[28] Ayers JW Poliak A Dredze M Leas EC Zhu Z Kelley JB . Comparing physician and artificial intelligence chatbot responses to patient questions posted to a public social media forum. JAMA Intern Med. (2023) 183:589–96. doi: 10.1001/jamainternmed.2023.1838 37115527 PMC10148230

[29] Fritsch SJ Blankenheim A Wahl A Hetfeld P Maassen O Deffge S . Attitudes and perception of artificial intelligence in healthcare: a cross-sectional survey among patients. Digit Health. (2022) 8:20552076221116772. doi: 10.1177/20552076221116772 35983102 PMC9380417

[30] Rojahn J Palu A Skiena S Jones JJ. American public opinion on artificial intelligence in healthcare. PLoS One. (2023) 18:e0294028. doi: 10.1371/journal.pone.0294028 37943752 PMC10635466

[31] Catapan SC Sazon H Zheng S Gallegos-Rejas V Mendis R Santiago PHR . A systematic review of consumers' and healthcare professionals' trust in digital healthcare. NPJ Digit Med. (2025) 8:115. doi: 10.1038/s41746-025-01510-8 39984678 PMC11845731

[32] Cheng C Beauchamp A Elsworth GR Osborne RH. Applying the electronic health literacy lens: systematic review of electronic health interventions targeted at socially disadvantaged groups. J Med Internet Res. (2020) 22:e18476. doi: 10.2196/18476 32788144 PMC7453328

[33] Wilson S Tolley C Mc Ardle R Lawson L Beswick E Hassan N . Recommendations to advance digital health equity: a systematic review of qualitative studies. NPJ Digit Med. (2024) 7:173. doi: 10.1038/s41746-024-01177-7 38951666 PMC11217442

[34] Oldhoff-Nuijsink C Derksen ME Engelsma T Peute LWP Fransen MP. Digital tools to support informed decision making among screening invitees in a vulnerable position for population-based cancer screening: a scoping review. Int J Med Inform. (2024) 192:105625. doi: 10.1016/j.ijmedinf.2024.105625 39317034

[35] Marko JGO Neagu CD Anand PB. Examining inclusivity: the use of AI and diverse populations in health and social care. BMC Med Inform Decis Mak. (2025) 25:57. doi: 10.1186/s12911-025-02884-1 39910518 PMC11796235

[36] Bundesministerium der Justiz. Sozialgesetzbuch Fünftes Buch (SGB V) § 20k Förderung der digitalen Gesundheitskompetenz. Berlin: Bundesministerium der Justiz; [cited 2026 Jun 8]. Available online at: https://www.gesetze-im-internet.de/sgb_5/__20k.html

[37] Wang L Wan Z Ni C Song Q Li Y Clayton E . Applications and concerns of ChatGPT and other conversational large language models in health care: systematic review. J Med Internet Res. (2024) 26:e22769. doi: 10.2196/22769 39509695 PMC11582494

